# Methyl 1-cyclo­hexyl-6,7-dimeth­oxy-3,4-dihydro­isoquinoline-3-carboxyl­ate

**DOI:** 10.1107/S1600536811009032

**Published:** 2011-03-15

**Authors:** Tricia Naicker, Thavendran Govender, Hendrik. G Kruger, Glenn. E. M Maguire

**Affiliations:** a School of Pharmacy and Pharmacology, University of KwaZulu-Natal, Durban 4000, South Africa; b School of Chemistry, University of KwaZulu-Natal, Durban 4000, South Africa

## Abstract

There are two independent mol­ecules in the asymmetric unit of the title compound, C_19_H_25_NO_4_. A single C—H⋯π inter­action and various inter­molecular contacts (2.65–2.83 Å) link the independent mol­ecules in the crystal structure. The N-containing six-membered ring assumes a twisted half-boat conformation.

## Related literature

For related structures, see: Naicker *et al.* (2010*a* ▶,*b* ▶, 2011 ▶).
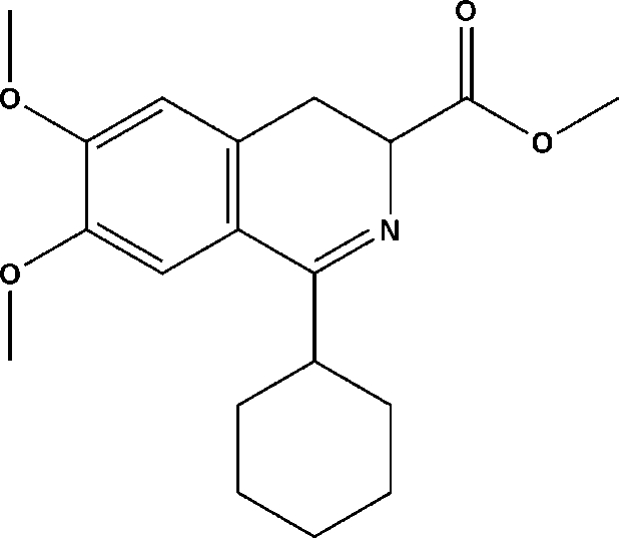


## Experimental

### 

#### Crystal data

C_19_H_25_NO_4_
*M* *_r_* = 331.40
Triclinic, 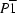

*a* = 9.5720 (2) Å
*b* = 10.8441 (4) Å
*c* = 17.5925 (6) Å
α = 80.941 (1)°
β = 75.267 (2)°
γ = 89.343 (2)°
*V* = 1743.25 (9) Å^3^
*Z* = 4
Mo *K*α radiation
μ = 0.09 mm^−1^
*T* = 173 K
0.44 × 0.38 × 0.35 mm

#### Data collection

Bruker Kappa DUO APEXII diffractometer
15272 measured reflections
7670 independent reflections
6167 reflections with *I* > 2σ(*I*)
*R*_int_ = 0.014

#### Refinement

*R*[*F* ^2^ > 2σ(*F* ^2^)] = 0.040
*wR*(*F* ^2^) = 0.110
*S* = 1.05
7670 reflections
433 parameters
H-atom parameters constrained
Δρ_max_ = 0.31 e Å^−3^
Δρ_min_ = −0.27 e Å^−3^

### 

Data collection: *APEX2* (Bruker, 2006 ▶); cell refinement: *SAINT* (Bruker, 2006 ▶); data reduction: *SAINT*; program(s) used to solve structure: *SHELXS97* (Sheldrick, 2008 ▶); program(s) used to refine structure: *SHELXL97* (Sheldrick, 2008 ▶); molecular graphics: *OLEX2* (Dolomanov *et al.*, 2009 ▶); software used to prepare material for publication: *SHELXL97*.

## Supplementary Material

Crystal structure: contains datablocks I, global. DOI: 10.1107/S1600536811009032/hg5002sup1.cif

Structure factors: contains datablocks I. DOI: 10.1107/S1600536811009032/hg5002Isup2.hkl

Additional supplementary materials:  crystallographic information; 3D view; checkCIF report

## Figures and Tables

**Table 1:** Hydrogen-bond geometry (Å, °) *Cg* is the centroid of the C2*B*–C7*B* ring.

*D*—H⋯*A*	*D*—H	H⋯*A*	*D*⋯*A*	*D*—H⋯*A*
C8*A*—H8*A*1⋯*Cg*^i^	0.99	2.96	3.9272 (13)	167

Symmetry code: (i) 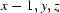.

**Fig. 1.**
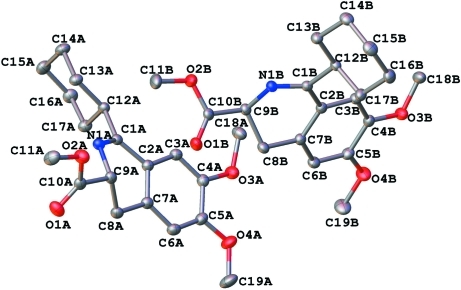
The molecular structure of the title compound with the atom numbering scheme. Displacement ellipsoids are drawn at the 40% probability level. Hydrogen atoms have been omitted for clarity.

**Fig. 2.**
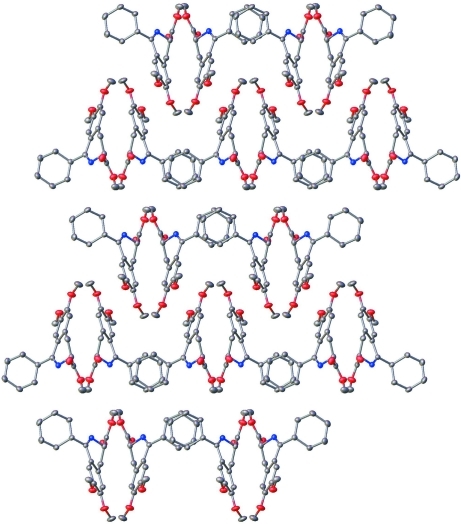
A partial projection of the title compound, viewed along the [100] plane.

## supplementary crystallographic information

### Comment

The title compound is a precursor in the synthesis of novel chiral catalysts containing a tetrahydroisoquinoline framework. Upon oxidation of the sp2 hybridized nitrogen, the oxide form of this compound and derivatives are currently being tested in our laboratory as novel organocatalysts for asymmetric allylation reactions (Naicker *et al.* 2010*a*).

The structure has triclinic (*P*1) symmetry with two molecules in the asymmetric unit (Fig. 1). These two molecules are linked by various intermolecular short contact interactions and one C—H··· π (C2B—C7B ring) bond (Table 1). The crystal packing reveals that *via* the centre of symmetry operation the enantiomer generates its mirror image. This results in a layered packing along the *a* axis (Fig. 2).

From the crystal structure it is evident that the *N*-containing six membered ring assumes a twisted half boat conformation (Fig. 1). This heterocyclic ring within similar structures displays either a half chair ((Naicker *et al.* 2010*b*) or half boat (Naicker *et al.* 2011) conformation. A possible reason for the change is the introduction of the sp2 hybrized nitrogen atom.

As anticipated the cyclohexane moieties adopt chair conformations.

### Experimental

To a solution of methyl 2-(cyclohexanecarboxamido)-3-(3,4-dimethoxyphenyl)propanoate (0.30 g, 0.86 mmol) in toluene (20 ml), phosphoryl trichloride (8.7 eq, 0.68 ml) was added. The mixture was refluxed for 4 h. Thereafter the toluene was evapourated under reduced pressure and the resulting residue treated with aqueous saturated potassium carbonate (15 ml) and extracted with ethyl acetate (2 *x* 10 ml). The organic extracts were combined and dried over anhydrous magnesium sulfate and then concentrated to dryness affording the crude product which was purified by column chromatography, (1:1 ethyl acetate, hexane), *R*_f_ = 0.5. Recrystallization from chloroform at room temperrature afforded colourless crystals suitable for X-ray analysis.

Melting point 377–379 K.

IR (neat) ν_max_: 2928, 1738, 1514, 1249, 1149, 752 cm^-1^.

^1^H NMR (400 MHz, CDCl_3_) δ 7.05 (s, 1H), 6.70 (s, 1H), 4.29 (t, *J* = 8.4 Hz, 1H), 3.91 (s, 6H), 3.75 (s, 3H), 2.87 (m, 2H), 1.87–1.21 (m, 11H).

^13^C NMR (101 MHz, CDCl_3_) δ 173.57, 151.04, 147.77, 129.82, 121.36, 110.60, 108.80, 59.40, 56.33, 55.98, 52.25, 42.67, 31.44, 30.80, 28.55, 26.55, 26.42, 26.14.

### Refinement

All non-hydrogen atoms were refined anisotropically. The hydrogen atoms were placed in idealized positions in a riding model with *U*_iso_ set at 1.2 or 1.5 times those of their parent atoms (1.2 for tertiary C—H, secondary C—H2 and aromatic C—H or N—H groups and 1.5 for methyl C—H3) and fixed C—H bond lengths (*e.g.* 0.88 Å for N—H and others ranging from 0.95 Å to 1.00 Å).

### Crystal data

**Table:**

C_19_H_25_NO_4_	*Z* = 4
*M**_r_* = 331.40	*F*(000) = 712
Triclinic, *P*1	*D*_x_ = 1.263 Mg m^−^^3^
Hall symbol: -P 1	Melting point: 377 K
*a* = 9.5720 (2) Å	Mo *K*α radiation, λ = 0.71073 Å
*b* = 10.8441 (4) Å	Cell parameters from 15272 reflections
*c* = 17.5925 (6) Å	θ = 2.2–27.1°
α = 80.941 (1)°	µ = 0.09 mm^−^^1^
β = 75.267 (2)°	*T* = 173 K
γ = 89.343 (2)°	Block, colourless
*V* = 1743.25 (9) Å^3^	0.44 × 0.38 × 0.35 mm

### Data collection

**Table:**

Bruker Kappa DUO APEXII diffractometer	6167 reflections with *I* > 2σ(*I*)
Radiation source: fine-focus sealed tube	*R*_int_ = 0.014
graphite	θ_max_ = 27.1°, θ_min_ = 2.2°
1.2° φ scans and ω	*h* = −12→12
15272 measured reflections	*k* = −13→13
7670 independent reflections	*l* = −22→22

### Refinement

**Table:**

Refinement on *F*^2^	Primary atom site location: structure-invariant direct methods
Least-squares matrix: full	Secondary atom site location: difference Fourier map
*R*[*F*^2^ > 2σ(*F*^2^)] = 0.040	Hydrogen site location: inferred from neighbouring sites
*wR*(*F*^2^) = 0.110	H-atom parameters constrained
*S* = 1.05	*w* = 1/[σ^2^(*F*_o_^2^) + (0.0577*P*)^2^ + 0.3824*P*] where *P* = (*F*_o_^2^ + 2*F*_c_^2^)/3
7670 reflections	(Δ/σ)_max_ < 0.001
433 parameters	Δρ_max_ = 0.31 e Å^−^^3^
0 restraints	Δρ_min_ = −0.27 e Å^−^^3^

### Special details

**Table:**

Experimental. Half sphere of data collected using *SAINT* strategy (Bruker, 2006). 2000). Crystal to detector distance = 30 mm; combination of φ and ω scans of 1.0°, 20 s per °, 2 iterations.
Geometry. All e.s.d.'s (except the e.s.d. in the dihedral angle between two l.s. planes) are estimated using the full covariance matrix. The cell e.s.d.'s are taken into account individually in the estimation of e.s.d.'s in distances, angles and torsion angles; correlations between e.s.d.'s in cell parameters are only used when they are defined by crystal symmetry. An approximate (isotropic) treatment of cell e.s.d.'s is used for estimating e.s.d.'s involving l.s. planes.
Refinement. Refinement of *F*^2^ against ALL reflections. The weighted *R*-factor *wR* and goodness of fit *S* are based on *F*^2^, conventional *R*-factors *R* are based on *F*, with *F* set to zero for negative *F*^2^. The threshold expression of *F*^2^ > σ(*F*^2^) is used only for calculating *R*-factors(gt) *etc*. and is not relevant to the choice of reflections for refinement. *R*-factors based on *F*^2^ are statistically about twice as large as those based on *F*, and *R*- factors based on ALL data will be even larger.

### Fractional atomic coordinates and isotropic or equivalent isotropic displacement parameters (Å^2^)

**Table:**

	*x*	*y*	*z*	*U*_iso_*/*U*_eq_	
O1A	0.26686 (9)	0.71209 (8)	0.19855 (6)	0.0351 (2)	
O2A	0.44184 (10)	0.82229 (9)	0.10407 (5)	0.0347 (2)	
O3A	0.92893 (9)	0.57579 (9)	0.43538 (5)	0.0334 (2)	
O4A	0.69445 (10)	0.64633 (10)	0.52482 (5)	0.0390 (2)	
N1A	0.60340 (10)	0.63179 (9)	0.17689 (6)	0.0232 (2)	
C1A	0.69095 (12)	0.57660 (10)	0.21465 (7)	0.0217 (2)	
C2A	0.69670 (12)	0.59983 (10)	0.29469 (7)	0.0225 (2)	
C3A	0.81857 (13)	0.57647 (11)	0.32469 (7)	0.0246 (2)	
H3A	0.9041	0.5485	0.2920	0.029*	
C4A	0.81548 (13)	0.59380 (11)	0.40118 (7)	0.0259 (3)	
C5A	0.68716 (14)	0.63287 (11)	0.45005 (7)	0.0277 (3)	
C6A	0.56750 (13)	0.65742 (11)	0.42009 (7)	0.0280 (3)	
H6A	0.4816	0.6846	0.4529	0.034*	
C7A	0.57196 (13)	0.64259 (11)	0.34225 (7)	0.0241 (2)	
C8A	0.44615 (13)	0.67041 (11)	0.30585 (7)	0.0262 (3)	
H8A1	0.3839	0.7326	0.3326	0.031*	
H8A2	0.3873	0.5933	0.3120	0.031*	
C9A	0.50709 (12)	0.72152 (11)	0.21826 (7)	0.0238 (2)	
H9A	0.5639	0.8004	0.2136	0.029*	
C10A	0.39021 (13)	0.74971 (11)	0.17448 (7)	0.0247 (3)	
C11A	0.34173 (15)	0.84768 (14)	0.05488 (8)	0.0380 (3)	
H11A	0.3897	0.9013	0.0049	0.057*	
H11B	0.2581	0.8901	0.0831	0.057*	
H11C	0.3095	0.7689	0.0432	0.057*	
C12A	0.78396 (12)	0.47538 (11)	0.17883 (7)	0.0228 (2)	
H12A	0.8855	0.4911	0.1810	0.027*	
C13A	0.78454 (13)	0.47279 (11)	0.09229 (7)	0.0263 (3)	
H13A	0.8243	0.5535	0.0595	0.032*	
H13B	0.6841	0.4620	0.0885	0.032*	
C14A	0.87518 (14)	0.36646 (12)	0.05994 (7)	0.0323 (3)	
H14A	0.8716	0.3659	0.0043	0.039*	
H14B	0.9771	0.3806	0.0600	0.039*	
C15A	0.81992 (15)	0.24052 (12)	0.10999 (8)	0.0338 (3)	
H15A	0.7213	0.2226	0.1057	0.041*	
H15B	0.8833	0.1740	0.0894	0.041*	
C16A	0.81694 (15)	0.24008 (12)	0.19685 (8)	0.0315 (3)	
H16A	0.9172	0.2467	0.2019	0.038*	
H16B	0.7734	0.1599	0.2287	0.038*	
C17A	0.73078 (13)	0.34755 (11)	0.22988 (7)	0.0266 (3)	
H17A	0.7387	0.3482	0.2848	0.032*	
H17B	0.6276	0.3339	0.2323	0.032*	
C18A	1.06746 (13)	0.56069 (14)	0.38401 (8)	0.0335 (3)	
H18A	1.1395	0.5492	0.4152	0.050*	
H18B	1.0938	0.6352	0.3433	0.050*	
H18C	1.0642	0.4873	0.3583	0.050*	
C19A	0.56717 (17)	0.68754 (16)	0.57547 (8)	0.0463 (4)	
H19A	0.5848	0.6939	0.6272	0.069*	
H19B	0.4873	0.6274	0.5829	0.069*	
H19C	0.5421	0.7695	0.5510	0.069*	
O1B	0.76423 (9)	0.92685 (9)	0.20742 (5)	0.0338 (2)	
O2B	0.94374 (9)	0.88901 (9)	0.10537 (5)	0.0351 (2)	
O3B	1.43063 (9)	0.95138 (9)	0.43879 (5)	0.0307 (2)	
O4B	1.19740 (10)	0.83840 (9)	0.52876 (5)	0.0363 (2)	
N1B	1.10515 (10)	1.02495 (9)	0.18028 (6)	0.0216 (2)	
C1B	1.19338 (12)	1.05972 (10)	0.21725 (7)	0.0203 (2)	
C2B	1.19881 (12)	0.99731 (10)	0.29783 (7)	0.0212 (2)	
C3B	1.32033 (12)	1.00537 (11)	0.32793 (7)	0.0230 (2)	
H3B	1.4054	1.0488	0.2951	0.028*	
C4B	1.31746 (13)	0.95079 (11)	0.40472 (7)	0.0239 (2)	
C5B	1.18998 (13)	0.88816 (11)	0.45380 (7)	0.0257 (3)	
C6B	1.07080 (13)	0.87890 (11)	0.42371 (7)	0.0255 (3)	
H6B	0.9854	0.8360	0.4566	0.031*	
C7B	1.07523 (12)	0.93198 (10)	0.34560 (7)	0.0221 (2)	
C8B	0.94865 (12)	0.92380 (11)	0.30996 (7)	0.0239 (2)	
H8B1	0.8886	0.9982	0.3167	0.029*	
H8B2	0.8878	0.8484	0.3369	0.029*	
C9B	1.00823 (12)	0.91704 (11)	0.22208 (7)	0.0219 (2)	
H9B	1.0649	0.8394	0.2173	0.026*	
C10B	0.89023 (12)	0.91202 (10)	0.17937 (7)	0.0224 (2)	
C11B	0.84171 (15)	0.88618 (14)	0.05758 (8)	0.0376 (3)	
H11D	0.8925	0.8688	0.0047	0.056*	
H11E	0.7958	0.9672	0.0523	0.056*	
H11F	0.7676	0.8206	0.0834	0.056*	
C12B	1.28788 (12)	1.17590 (10)	0.17986 (6)	0.0215 (2)	
H12B	1.3883	1.1575	0.1844	0.026*	
C13B	1.29372 (13)	1.21677 (11)	0.09197 (7)	0.0254 (3)	
H13C	1.3352	1.1496	0.0617	0.030*	
H13D	1.1944	1.2304	0.0859	0.030*	
C14B	1.38490 (14)	1.33662 (12)	0.05783 (7)	0.0296 (3)	
H14C	1.4859	1.3214	0.0604	0.036*	
H14D	1.3850	1.3615	0.0012	0.036*	
C15B	1.32624 (15)	1.44174 (12)	0.10374 (8)	0.0335 (3)	
H15C	1.3896	1.5173	0.0819	0.040*	
H15D	1.2285	1.4621	0.0971	0.040*	
C16B	1.31858 (14)	1.40480 (12)	0.19170 (8)	0.0315 (3)	
H16C	1.2729	1.4720	0.2208	0.038*	
H16D	1.4178	1.3955	0.1987	0.038*	
C17B	1.23242 (13)	1.28270 (11)	0.22711 (7)	0.0265 (3)	
H17C	1.1296	1.2961	0.2279	0.032*	
H17D	1.2382	1.2580	0.2828	0.032*	
C18B	1.56838 (13)	0.99195 (14)	0.38721 (7)	0.0321 (3)	
H18D	1.6404	0.9881	0.4183	0.048*	
H18E	1.5631	1.0781	0.3612	0.048*	
H18F	1.5962	0.9375	0.3467	0.048*	
C19B	1.07091 (17)	0.77248 (15)	0.57943 (8)	0.0439 (4)	
H19D	1.0882	0.7411	0.6315	0.066*	
H19E	1.0481	0.7021	0.5554	0.066*	
H19F	0.9896	0.8290	0.5861	0.066*	

### Atomic displacement parameters (Å^2^)

**Table:**

	*U*^11^	*U*^22^	*U*^33^	*U*^12^	*U*^13^	*U*^23^
O1A	0.0238 (5)	0.0336 (5)	0.0472 (6)	0.0030 (4)	−0.0116 (4)	−0.0005 (4)
O2A	0.0292 (5)	0.0439 (6)	0.0305 (5)	0.0037 (4)	−0.0111 (4)	0.0005 (4)
O3A	0.0270 (5)	0.0492 (6)	0.0246 (5)	0.0019 (4)	−0.0088 (4)	−0.0040 (4)
O4A	0.0411 (5)	0.0547 (6)	0.0238 (5)	0.0090 (5)	−0.0089 (4)	−0.0134 (4)
N1A	0.0210 (5)	0.0224 (5)	0.0256 (5)	0.0019 (4)	−0.0049 (4)	−0.0038 (4)
C1A	0.0195 (5)	0.0215 (6)	0.0225 (6)	−0.0007 (4)	−0.0031 (4)	−0.0026 (4)
C2A	0.0239 (6)	0.0204 (6)	0.0227 (6)	0.0008 (4)	−0.0051 (5)	−0.0029 (4)
C3A	0.0237 (6)	0.0248 (6)	0.0241 (6)	0.0017 (5)	−0.0044 (5)	−0.0032 (5)
C4A	0.0270 (6)	0.0255 (6)	0.0252 (6)	−0.0008 (5)	−0.0086 (5)	−0.0010 (5)
C5A	0.0344 (7)	0.0273 (6)	0.0215 (6)	0.0009 (5)	−0.0067 (5)	−0.0045 (5)
C6A	0.0286 (6)	0.0272 (6)	0.0260 (6)	0.0037 (5)	−0.0024 (5)	−0.0058 (5)
C7A	0.0247 (6)	0.0212 (6)	0.0255 (6)	0.0019 (4)	−0.0052 (5)	−0.0037 (5)
C8A	0.0222 (6)	0.0275 (6)	0.0289 (6)	0.0043 (5)	−0.0045 (5)	−0.0080 (5)
C9A	0.0224 (6)	0.0209 (6)	0.0288 (6)	0.0021 (4)	−0.0069 (5)	−0.0054 (5)
C10A	0.0251 (6)	0.0192 (6)	0.0311 (6)	0.0056 (4)	−0.0075 (5)	−0.0074 (5)
C11A	0.0378 (8)	0.0496 (9)	0.0302 (7)	0.0153 (6)	−0.0151 (6)	−0.0076 (6)
C12A	0.0200 (5)	0.0254 (6)	0.0234 (6)	0.0035 (4)	−0.0059 (4)	−0.0051 (5)
C13A	0.0285 (6)	0.0273 (6)	0.0217 (6)	0.0034 (5)	−0.0043 (5)	−0.0032 (5)
C14A	0.0352 (7)	0.0352 (7)	0.0253 (6)	0.0065 (6)	−0.0030 (5)	−0.0096 (5)
C15A	0.0390 (7)	0.0289 (7)	0.0353 (7)	0.0073 (5)	−0.0083 (6)	−0.0126 (6)
C16A	0.0340 (7)	0.0261 (6)	0.0349 (7)	0.0088 (5)	−0.0099 (6)	−0.0051 (5)
C17A	0.0292 (6)	0.0260 (6)	0.0237 (6)	0.0055 (5)	−0.0060 (5)	−0.0026 (5)
C18A	0.0246 (6)	0.0451 (8)	0.0285 (7)	−0.0013 (5)	−0.0068 (5)	0.0011 (6)
C19A	0.0537 (9)	0.0590 (10)	0.0279 (7)	0.0144 (8)	−0.0072 (6)	−0.0183 (7)
O1B	0.0220 (5)	0.0456 (6)	0.0363 (5)	−0.0022 (4)	−0.0075 (4)	−0.0139 (4)
O2B	0.0273 (5)	0.0546 (6)	0.0272 (5)	−0.0014 (4)	−0.0088 (4)	−0.0142 (4)
O3B	0.0245 (4)	0.0466 (5)	0.0217 (4)	−0.0005 (4)	−0.0082 (3)	−0.0035 (4)
O4B	0.0373 (5)	0.0481 (6)	0.0204 (4)	−0.0070 (4)	−0.0091 (4)	0.0068 (4)
N1B	0.0198 (5)	0.0214 (5)	0.0228 (5)	−0.0009 (4)	−0.0046 (4)	−0.0026 (4)
C1B	0.0195 (5)	0.0203 (5)	0.0202 (5)	0.0011 (4)	−0.0035 (4)	−0.0029 (4)
C2B	0.0229 (6)	0.0194 (5)	0.0210 (6)	0.0002 (4)	−0.0052 (4)	−0.0030 (4)
C3B	0.0232 (6)	0.0232 (6)	0.0214 (6)	−0.0014 (4)	−0.0043 (4)	−0.0023 (4)
C4B	0.0241 (6)	0.0260 (6)	0.0231 (6)	0.0014 (5)	−0.0076 (5)	−0.0057 (5)
C5B	0.0314 (6)	0.0261 (6)	0.0187 (6)	0.0000 (5)	−0.0064 (5)	−0.0010 (5)
C6B	0.0262 (6)	0.0248 (6)	0.0226 (6)	−0.0042 (5)	−0.0029 (5)	−0.0004 (5)
C7B	0.0238 (6)	0.0198 (5)	0.0224 (6)	−0.0002 (4)	−0.0053 (4)	−0.0030 (4)
C8B	0.0213 (6)	0.0247 (6)	0.0241 (6)	−0.0033 (4)	−0.0045 (5)	−0.0006 (5)
C9B	0.0210 (5)	0.0200 (5)	0.0245 (6)	−0.0009 (4)	−0.0058 (4)	−0.0028 (4)
C10B	0.0240 (6)	0.0176 (5)	0.0251 (6)	−0.0031 (4)	−0.0059 (5)	−0.0024 (4)
C11B	0.0383 (8)	0.0505 (9)	0.0275 (7)	−0.0101 (6)	−0.0141 (6)	−0.0060 (6)
C12B	0.0202 (5)	0.0228 (6)	0.0208 (6)	−0.0030 (4)	−0.0059 (4)	0.0002 (4)
C13B	0.0267 (6)	0.0279 (6)	0.0202 (6)	−0.0032 (5)	−0.0055 (5)	−0.0008 (5)
C14B	0.0294 (6)	0.0319 (7)	0.0235 (6)	−0.0041 (5)	−0.0046 (5)	0.0049 (5)
C15B	0.0352 (7)	0.0241 (6)	0.0370 (7)	−0.0039 (5)	−0.0072 (6)	0.0047 (5)
C16B	0.0344 (7)	0.0245 (6)	0.0353 (7)	−0.0063 (5)	−0.0082 (6)	−0.0047 (5)
C17B	0.0293 (6)	0.0256 (6)	0.0233 (6)	−0.0057 (5)	−0.0044 (5)	−0.0037 (5)
C18B	0.0230 (6)	0.0485 (8)	0.0256 (6)	0.0012 (5)	−0.0060 (5)	−0.0087 (6)
C19B	0.0484 (9)	0.0524 (9)	0.0244 (7)	−0.0128 (7)	−0.0077 (6)	0.0116 (6)

### Geometric parameters (Å, °)

**Table:**

O1A—C10A	1.2009 (15)	O1B—C10B	1.2008 (14)
O2A—C10A	1.3373 (15)	O2B—C10B	1.3340 (14)
O2A—C11A	1.4436 (15)	O2B—C11B	1.4449 (15)
O3A—C4A	1.3674 (14)	O3B—C4B	1.3654 (14)
O3A—C18A	1.4262 (15)	O3B—C18B	1.4263 (15)
O4A—C5A	1.3652 (15)	O4B—C5B	1.3631 (14)
O4A—C19A	1.4278 (17)	O4B—C19B	1.4289 (16)
N1A—C1A	1.2838 (15)	N1B—C1B	1.2815 (14)
N1A—C9A	1.4790 (14)	N1B—C9B	1.4757 (14)
C1A—C2A	1.4835 (16)	C1B—C2B	1.4849 (16)
C1A—C12A	1.5181 (15)	C1B—C12B	1.5168 (15)
C2A—C7A	1.3936 (16)	C2B—C7B	1.3905 (16)
C2A—C3A	1.4028 (16)	C2B—C3B	1.4040 (16)
C3A—C4A	1.3807 (17)	C3B—C4B	1.3806 (16)
C3A—H3A	0.9500	C3B—H3B	0.9500
C4A—C5A	1.4117 (17)	C4B—C5B	1.4122 (16)
C5A—C6A	1.3843 (18)	C5B—C6B	1.3851 (17)
C6A—C7A	1.3937 (17)	C6B—C7B	1.3941 (16)
C6A—H6A	0.9500	C6B—H6B	0.9500
C7A—C8A	1.5081 (16)	C7B—C8B	1.5085 (16)
C8A—C9A	1.5176 (17)	C8B—C9B	1.5188 (16)
C8A—H8A1	0.9900	C8B—H8B1	0.9900
C8A—H8A2	0.9900	C8B—H8B2	0.9900
C9A—C10A	1.5148 (16)	C9B—C10B	1.5130 (16)
C9A—H9A	1.0000	C9B—H9B	1.0000
C11A—H11A	0.9800	C11B—H11D	0.9800
C11A—H11B	0.9800	C11B—H11E	0.9800
C11A—H11C	0.9800	C11B—H11F	0.9800
C12A—C13A	1.5257 (16)	C12B—C13B	1.5268 (15)
C12A—C17A	1.5424 (16)	C12B—C17B	1.5423 (16)
C12A—H12A	1.0000	C12B—H12B	1.0000
C13A—C14A	1.5293 (17)	C13B—C14B	1.5248 (16)
C13A—H13A	0.9900	C13B—H13C	0.9900
C13A—H13B	0.9900	C13B—H13D	0.9900
C14A—C15A	1.5235 (19)	C14B—C15B	1.5204 (18)
C14A—H14A	0.9900	C14B—H14C	0.9900
C14A—H14B	0.9900	C14B—H14D	0.9900
C15A—C16A	1.5204 (18)	C15B—C16B	1.5193 (18)
C15A—H15A	0.9900	C15B—H15C	0.9900
C15A—H15B	0.9900	C15B—H15D	0.9900
C16A—C17A	1.5243 (16)	C16B—C17B	1.5249 (16)
C16A—H16A	0.9900	C16B—H16C	0.9900
C16A—H16B	0.9900	C16B—H16D	0.9900
C17A—H17A	0.9900	C17B—H17C	0.9900
C17A—H17B	0.9900	C17B—H17D	0.9900
C18A—H18A	0.9800	C18B—H18D	0.9800
C18A—H18B	0.9800	C18B—H18E	0.9800
C18A—H18C	0.9800	C18B—H18F	0.9800
C19A—H19A	0.9800	C19B—H19D	0.9800
C19A—H19B	0.9800	C19B—H19E	0.9800
C19A—H19C	0.9800	C19B—H19F	0.9800
			
C10A—O2A—C11A	115.80 (10)	C10B—O2B—C11B	116.42 (10)
C4A—O3A—C18A	117.12 (9)	C4B—O3B—C18B	117.00 (9)
C5A—O4A—C19A	116.57 (11)	C5B—O4B—C19B	116.46 (10)
C1A—N1A—C9A	115.69 (10)	C1B—N1B—C9B	116.15 (9)
N1A—C1A—C2A	122.95 (10)	N1B—C1B—C2B	122.85 (10)
N1A—C1A—C12A	118.45 (10)	N1B—C1B—C12B	118.43 (10)
C2A—C1A—C12A	118.40 (10)	C2B—C1B—C12B	118.53 (9)
C7A—C2A—C3A	119.61 (11)	C7B—C2B—C3B	119.51 (10)
C7A—C2A—C1A	117.24 (10)	C7B—C2B—C1B	117.55 (10)
C3A—C2A—C1A	123.11 (10)	C3B—C2B—C1B	122.90 (10)
C4A—C3A—C2A	120.67 (11)	C4B—C3B—C2B	120.68 (11)
C4A—C3A—H3A	119.7	C4B—C3B—H3B	119.7
C2A—C3A—H3A	119.7	C2B—C3B—H3B	119.7
O3A—C4A—C3A	125.14 (11)	O3B—C4B—C3B	125.04 (11)
O3A—C4A—C5A	115.40 (10)	O3B—C4B—C5B	115.45 (10)
C3A—C4A—C5A	119.46 (11)	C3B—C4B—C5B	119.51 (11)
O4A—C5A—C6A	125.05 (11)	O4B—C5B—C6B	125.12 (11)
O4A—C5A—C4A	115.09 (11)	O4B—C5B—C4B	115.13 (10)
C6A—C5A—C4A	119.83 (11)	C6B—C5B—C4B	119.74 (11)
C5A—C6A—C7A	120.57 (11)	C5B—C6B—C7B	120.51 (11)
C5A—C6A—H6A	119.7	C5B—C6B—H6B	119.7
C7A—C6A—H6A	119.7	C7B—C6B—H6B	119.7
C2A—C7A—C6A	119.80 (11)	C2B—C7B—C6B	120.00 (11)
C2A—C7A—C8A	117.26 (10)	C2B—C7B—C8B	117.49 (10)
C6A—C7A—C8A	122.94 (11)	C6B—C7B—C8B	122.51 (10)
C7A—C8A—C9A	107.65 (9)	C7B—C8B—C9B	107.78 (9)
C7A—C8A—H8A1	110.2	C7B—C8B—H8B1	110.2
C9A—C8A—H8A1	110.2	C9B—C8B—H8B1	110.2
C7A—C8A—H8A2	110.2	C7B—C8B—H8B2	110.2
C9A—C8A—H8A2	110.2	C9B—C8B—H8B2	110.2
H8A1—C8A—H8A2	108.5	H8B1—C8B—H8B2	108.5
N1A—C9A—C10A	106.68 (9)	N1B—C9B—C10B	107.54 (9)
N1A—C9A—C8A	111.50 (9)	N1B—C9B—C8B	111.99 (9)
C10A—C9A—C8A	112.55 (10)	C10B—C9B—C8B	112.49 (9)
N1A—C9A—H9A	108.7	N1B—C9B—H9B	108.2
C10A—C9A—H9A	108.7	C10B—C9B—H9B	108.2
C8A—C9A—H9A	108.7	C8B—C9B—H9B	108.2
O1A—C10A—O2A	123.43 (11)	O1B—C10B—O2B	123.52 (11)
O1A—C10A—C9A	125.32 (11)	O1B—C10B—C9B	125.18 (11)
O2A—C10A—C9A	111.24 (10)	O2B—C10B—C9B	111.29 (10)
O2A—C11A—H11A	109.5	O2B—C11B—H11D	109.5
O2A—C11A—H11B	109.5	O2B—C11B—H11E	109.5
H11A—C11A—H11B	109.5	H11D—C11B—H11E	109.5
O2A—C11A—H11C	109.5	O2B—C11B—H11F	109.5
H11A—C11A—H11C	109.5	H11D—C11B—H11F	109.5
H11B—C11A—H11C	109.5	H11E—C11B—H11F	109.5
C1A—C12A—C13A	113.27 (9)	C1B—C12B—C13B	113.24 (9)
C1A—C12A—C17A	108.99 (9)	C1B—C12B—C17B	109.00 (9)
C13A—C12A—C17A	109.99 (10)	C13B—C12B—C17B	110.04 (9)
C1A—C12A—H12A	108.1	C1B—C12B—H12B	108.1
C13A—C12A—H12A	108.1	C13B—C12B—H12B	108.1
C17A—C12A—H12A	108.1	C17B—C12B—H12B	108.1
C12A—C13A—C14A	111.25 (10)	C14B—C13B—C12B	111.38 (10)
C12A—C13A—H13A	109.4	C14B—C13B—H13C	109.4
C14A—C13A—H13A	109.4	C12B—C13B—H13C	109.4
C12A—C13A—H13B	109.4	C14B—C13B—H13D	109.4
C14A—C13A—H13B	109.4	C12B—C13B—H13D	109.4
H13A—C13A—H13B	108.0	H13C—C13B—H13D	108.0
C15A—C14A—C13A	111.17 (10)	C15B—C14B—C13B	111.03 (10)
C15A—C14A—H14A	109.4	C15B—C14B—H14C	109.4
C13A—C14A—H14A	109.4	C13B—C14B—H14C	109.4
C15A—C14A—H14B	109.4	C15B—C14B—H14D	109.4
C13A—C14A—H14B	109.4	C13B—C14B—H14D	109.4
H14A—C14A—H14B	108.0	H14C—C14B—H14D	108.0
C16A—C15A—C14A	110.90 (11)	C16B—C15B—C14B	110.97 (10)
C16A—C15A—H15A	109.5	C16B—C15B—H15C	109.4
C14A—C15A—H15A	109.5	C14B—C15B—H15C	109.4
C16A—C15A—H15B	109.5	C16B—C15B—H15D	109.4
C14A—C15A—H15B	109.5	C14B—C15B—H15D	109.4
H15A—C15A—H15B	108.0	H15C—C15B—H15D	108.0
C15A—C16A—C17A	111.81 (10)	C15B—C16B—C17B	111.83 (10)
C15A—C16A—H16A	109.3	C15B—C16B—H16C	109.2
C17A—C16A—H16A	109.3	C17B—C16B—H16C	109.2
C15A—C16A—H16B	109.3	C15B—C16B—H16D	109.3
C17A—C16A—H16B	109.3	C17B—C16B—H16D	109.2
H16A—C16A—H16B	107.9	H16C—C16B—H16D	107.9
C16A—C17A—C12A	112.32 (10)	C16B—C17B—C12B	112.47 (10)
C16A—C17A—H17A	109.1	C16B—C17B—H17C	109.1
C12A—C17A—H17A	109.1	C12B—C17B—H17C	109.1
C16A—C17A—H17B	109.1	C16B—C17B—H17D	109.1
C12A—C17A—H17B	109.1	C12B—C17B—H17D	109.1
H17A—C17A—H17B	107.9	H17C—C17B—H17D	107.8
O3A—C18A—H18A	109.5	O3B—C18B—H18D	109.5
O3A—C18A—H18B	109.5	O3B—C18B—H18E	109.5
H18A—C18A—H18B	109.5	H18D—C18B—H18E	109.5
O3A—C18A—H18C	109.5	O3B—C18B—H18F	109.5
H18A—C18A—H18C	109.5	H18D—C18B—H18F	109.5
H18B—C18A—H18C	109.5	H18E—C18B—H18F	109.5
O4A—C19A—H19A	109.5	O4B—C19B—H19D	109.5
O4A—C19A—H19B	109.5	O4B—C19B—H19E	109.5
H19A—C19A—H19B	109.5	H19D—C19B—H19E	109.5
O4A—C19A—H19C	109.5	O4B—C19B—H19F	109.5
H19A—C19A—H19C	109.5	H19D—C19B—H19F	109.5
H19B—C19A—H19C	109.5	H19E—C19B—H19F	109.5
			
C9A—N1A—C1A—C2A	0.10 (16)	C9B—N1B—C1B—C2B	−0.58 (16)
C9A—N1A—C1A—C12A	174.91 (9)	C9B—N1B—C1B—C12B	−175.56 (9)
N1A—C1A—C2A—C7A	25.10 (16)	N1B—C1B—C2B—C7B	−23.57 (16)
C12A—C1A—C2A—C7A	−149.71 (11)	C12B—C1B—C2B—C7B	151.40 (10)
N1A—C1A—C2A—C3A	−157.28 (11)	N1B—C1B—C2B—C3B	158.64 (11)
C12A—C1A—C2A—C3A	27.91 (16)	C12B—C1B—C2B—C3B	−26.38 (16)
C7A—C2A—C3A—C4A	0.89 (17)	C7B—C2B—C3B—C4B	−0.99 (17)
C1A—C2A—C3A—C4A	−176.68 (11)	C1B—C2B—C3B—C4B	176.75 (10)
C18A—O3A—C4A—C3A	12.24 (18)	C18B—O3B—C4B—C3B	−11.50 (17)
C18A—O3A—C4A—C5A	−167.90 (11)	C18B—O3B—C4B—C5B	168.27 (11)
C2A—C3A—C4A—O3A	−178.80 (11)	C2B—C3B—C4B—O3B	178.65 (11)
C2A—C3A—C4A—C5A	1.35 (18)	C2B—C3B—C4B—C5B	−1.11 (17)
C19A—O4A—C5A—C6A	0.80 (19)	C19B—O4B—C5B—C6B	−0.35 (19)
C19A—O4A—C5A—C4A	179.10 (12)	C19B—O4B—C5B—C4B	−178.92 (12)
O3A—C4A—C5A—O4A	−0.41 (16)	O3B—C4B—C5B—O4B	0.79 (16)
C3A—C4A—C5A—O4A	179.45 (11)	C3B—C4B—C5B—O4B	−179.42 (10)
O3A—C4A—C5A—C6A	177.99 (11)	O3B—C4B—C5B—C6B	−177.86 (11)
C3A—C4A—C5A—C6A	−2.15 (18)	C3B—C4B—C5B—C6B	1.92 (18)
O4A—C5A—C6A—C7A	178.93 (11)	O4B—C5B—C6B—C7B	−179.14 (11)
C4A—C5A—C6A—C7A	0.70 (19)	C4B—C5B—C6B—C7B	−0.63 (18)
C3A—C2A—C7A—C6A	−2.34 (17)	C3B—C2B—C7B—C6B	2.29 (17)
C1A—C2A—C7A—C6A	175.37 (11)	C1B—C2B—C7B—C6B	−175.57 (10)
C3A—C2A—C7A—C8A	177.90 (10)	C3B—C2B—C7B—C8B	−178.78 (10)
C1A—C2A—C7A—C8A	−4.38 (15)	C1B—C2B—C7B—C8B	3.36 (15)
C5A—C6A—C7A—C2A	1.55 (18)	C5B—C6B—C7B—C2B	−1.49 (18)
C5A—C6A—C7A—C8A	−178.71 (11)	C5B—C6B—C7B—C8B	179.64 (11)
C2A—C7A—C8A—C9A	−34.63 (14)	C2B—C7B—C8B—C9B	34.32 (14)
C6A—C7A—C8A—C9A	145.63 (11)	C6B—C7B—C8B—C9B	−146.78 (11)
C1A—N1A—C9A—C10A	−165.90 (10)	C1B—N1B—C9B—C10B	165.87 (10)
C1A—N1A—C9A—C8A	−42.66 (13)	C1B—N1B—C9B—C8B	41.79 (13)
C7A—C8A—C9A—N1A	58.53 (12)	C7B—C8B—C9B—N1B	−57.19 (12)
C7A—C8A—C9A—C10A	178.37 (9)	C7B—C8B—C9B—C10B	−178.45 (9)
C11A—O2A—C10A—O1A	−3.78 (17)	C11B—O2B—C10B—O1B	1.87 (18)
C11A—O2A—C10A—C9A	175.80 (10)	C11B—O2B—C10B—C9B	−177.99 (10)
N1A—C9A—C10A—O1A	106.59 (13)	N1B—C9B—C10B—O1B	−115.11 (12)
C8A—C9A—C10A—O1A	−16.00 (16)	C8B—C9B—C10B—O1B	8.66 (16)
N1A—C9A—C10A—O2A	−72.97 (11)	N1B—C9B—C10B—O2B	64.74 (12)
C8A—C9A—C10A—O2A	164.44 (10)	C8B—C9B—C10B—O2B	−171.48 (10)
N1A—C1A—C12A—C13A	12.96 (15)	N1B—C1B—C12B—C13B	−15.49 (15)
C2A—C1A—C12A—C13A	−172.00 (10)	C2B—C1B—C12B—C13B	169.31 (10)
N1A—C1A—C12A—C17A	−109.84 (12)	N1B—C1B—C12B—C17B	107.35 (12)
C2A—C1A—C12A—C17A	65.21 (13)	C2B—C1B—C12B—C17B	−67.85 (13)
C1A—C12A—C13A—C14A	−177.95 (10)	C1B—C12B—C13B—C14B	177.73 (10)
C17A—C12A—C13A—C14A	−55.72 (13)	C17B—C12B—C13B—C14B	55.46 (13)
C12A—C13A—C14A—C15A	57.70 (14)	C12B—C13B—C14B—C15B	−57.98 (13)
C13A—C14A—C15A—C16A	−56.30 (14)	C13B—C14B—C15B—C16B	56.74 (14)
C14A—C15A—C16A—C17A	54.43 (15)	C14B—C15B—C16B—C17B	−54.43 (15)
C15A—C16A—C17A—C12A	−53.90 (14)	C15B—C16B—C17B—C12B	53.30 (14)
C1A—C12A—C17A—C16A	178.84 (10)	C1B—C12B—C17B—C16B	−178.07 (10)
C13A—C12A—C17A—C16A	54.10 (13)	C13B—C12B—C17B—C16B	−53.33 (13)

### Hydrogen-bond geometry (Å, °)

**Table:**

Cg is the centroid of the C2B–C7B ring.

**Table:**

*D*—H···*A*	*D*—H	H···*A*	*D*···*A*	*D*—H···*A*
C8A—H8A1···Cg^i^	0.99	2.96	3.9272 (13)	167

Symmetry codes: (i) *x*−1, *y*, *z*.
